# Top-down and bottom-up stimulation techniques combined with action observation treatment in stroke rehabilitation: a perspective

**DOI:** 10.3389/fneur.2023.1156987

**Published:** 2023-07-11

**Authors:** Fengxue Qi, Michael A. Nitsche, Xiping Ren, Duanwei Wang, Lijuan Wang

**Affiliations:** ^1^Sports, Exercise and Brain Sciences Laboratory, Beijing Sport University, Beijing, China; ^2^Department of Psychology and Neurosciences, Leibniz Research Centre for Working Environment and Human Factors, Dortmund, Germany; ^3^College of Physical Education and Health Sciences, Zhejiang Normal University, Jinhua, China; ^4^Shandong Mental Health Center, Shandong University, Jinan, Shandong, China; ^5^Key Laboratory of Exercise and Physical Fitness, Ministry of Education, Beijing Sport University, Beijing, China; ^6^School of Sports Medicine and Rehabilitation, Beijing Sport University, Beijing, China

**Keywords:** transcranial direct current stimulation, transcranial random noise stimulation, transcranial alternating current stimulation, transcranial magnetic stimulation, peripheral electrical stimulation, peripheral magnetic stimulation, action observation

## Abstract

Stroke is a central nervous system disease that causes structural lesions and functional impairments of the brain, resulting in varying types, and degrees of dysfunction. The bimodal balance-recovery model (interhemispheric competition model and vicariation model) has been proposed as the mechanism of functional recovery after a stroke. We analyzed how combinations of motor observation treatment approaches, transcranial electrical (TES) or magnetic (TMS) stimulation and peripheral electrical (PES) or magnetic (PMS) stimulation techniques can be taken as accessorial physical therapy methods on symptom reduction of stroke patients. We suggest that top-down and bottom-up stimulation techniques combined with action observation treatment synergistically might develop into valuable physical therapy strategies in neurorehabilitation after stroke. We explored how TES or TMS intervention over the contralesional hemisphere or the lesioned hemisphere combined with PES or PMS of the paretic limbs during motor observation followed by action execution have super-additive effects to potentiate the effect of conventional treatment in stroke patients. The proposed paradigm could be an innovative and adjunctive approach to potentiate the effect of conventional rehabilitation treatment, especially for those patients with severe motor deficits.

## Introduction

Stroke is a neurological syndrome caused by an acute vascular injury of the central nervous system. The syndrome incorporates the cerebral infarction, intracerebral hemorrhage, and subarachnoid hemorrhage (1). It is one of the primary causes of mortality and severe long-term disability. Among all causes of death, stroke ranks fifth following heart disease, cancer, chronic lower respiratory disease, and unintentional injuries/accidents (2). In 2019, the prevalence of stroke was 101 million cases and there were 6.55 million deaths in global (3). As a major concern of global health, stroke poses great social economic burden, for example, the overall expenses of stroke in US was $52.8 billion in 2017–2018, with mean direct expenses of $8,242 for each patient (4). Stroke ranks second among all the contributors to disability-adjust life-years globally (5). Long-term complications of stroke include pain syndromes, depression and anxiety, cognitive decline and dementia, as well as falls and fractures due to gait instability (2). Motor impairment of the contralateral limb (e.g., loss or limitation of muscle control, mobility, power, and dexterity) is one of the commonest and most detrimental consequences after stroke (6, 7). Dysfunctional motor control affects functional independence of activities of daily living, and thus reduces the quality of life.

Neurorehabilitation after a stroke includes multidisciplinary rehabilitation methods to compensate for the motor deficit, restore motor functions, and improve the life quality of patients (8, 9). Despite intensive therapeutic efforts during stroke rehabilitation, a relevant amount of stroke survivors failed to regain their motor functions that are important for activities of daily living completely (10). Therefore new/advanced approaches are required to optimize motor functions and reduce disability in stroke patients. Based on basic behavioral science and neuroscientific knowledge, novel rehabilitative approaches have been developed to ameliorate perceptual abilities and improve motor functions after stroke in the last few years (11, 12). These novel rehabilitative intervention modalities included action observation treatment (AOT), non-invasive brain stimulation (NIBS) as well as repetitive peripheral electrical or magnetic stimulation (13). These tools share features of targeted modulation of central nervous system activity, and neuroplasticity induction, and might hereby generate therapeutic benefits (11, 14). In this perspective paper, we aimed to discuss how combinations with these novel stimulation techniques and approaches can be taken as potential rehabilitation methods for stroke patients.

## Theoretical background and rationale

Brain structural damage of areas and connections, as well as inhibition of the ipsilesional primary motor and sensory cortex disrupts functional connectivity of the motor network and impairs functional network flexibility after stroke (15). A bimodal balance-recovery model has been proposed as the mechanism of functional recovery after a stroke. The extent of structural reserve of the lesioned hemisphere is related to functional reorganization and the involvement of the affected hemisphere in motor control (16). The interhemispheric competition model dominates in stroke patients with high structural reserve (less impairment) (16). Functional neuroimaging studies showed a dysbalance of motor cortex excitability in post-stroke, which is relative hypo-excitability in the ipsilesional hemisphere and hyper-excitability in the contralesional hemisphere (16–18). The hyperactive contralesional hemisphere inhibits cortical excitability of the ipsilesional hemisphere *via* transcallosal inhibition, and compromises motor output (19, 20). Based on the inter-hemispheric competition model, upregulating the excitability of the lesioned hemisphere and/or downregulating the excitability of the intact hemisphere may facilitate recovery in stroke patients (21). In patients with little structural reserve (more severe impairment), the vicariation model predicts stroke recovery. Activity in the contralesional hemisphere compensates for functional loss by the affected hemisphere (16). In this case, instead of predicting a worse outcome on the basis of the interhemispheric competition model, interhemispheric imbalance facilitates vicarious activity of the intact hemisphere, allowing substitutional plasticity (16). A recent longitudinal study by Lin et al. has verified this bimodal balance recovery hypothesis, indicating that the contralesional hemisphere modulates differently across chronic stroke patients with different levels of ipsilesional hemisphere reserve (22).

Neuroplasticity is an important physiological foundation for the neurorehabilitation of stroke patients. It refers to the life-long ability of the central nervous system for reorganization and adaptation, which includes strengthening and weakening synaptic connections, as well as the formation of new neural pathways. Neuroplasticity is a crucial foundation for learning and memory formation, and recovery of motor functions after neurological injuries (9). Modifying neural circuit function in response to external/environmental stimuli and subsequently affecting behavior, cognition, and motor function is a crucial property of the mammalian brain (23, 24). Functional plasticity and structural plasticity are two types of plasticity mechanisms (25). Functional plasticity refers to alterations in the strength of preexisting synaptic transmission, whereas structural plasticity incorporates the growth and deletion of synaptic connections (23, 25, 26). Synaptic plasticity can occur from the ultrastructure level to the brain network level along with short- and long-term alternations in Ca^2+^ dynamics, modulation of neurotransmission as well as expression of protein and gene (27). Synaptic plasticity is classified into Hebbian and homeostatic synaptic plasticity (25, 28). Hebbian synaptic plasticity is a positive feedback loop and unrestricted dynamics *via* strengthening (long-term potentiation, LTP) or weakening (long-term depression, LTD) of synaptic transmission (24, 26, 29). In contrast, homeostatic synaptic plasticity is a negative feedback loop and stabilized neural dynamics in which synaptic efficacy decreases in the case of high neuronal activities and increases when activities are low (25, 30). Animal studies largely contributed to our knowledge about physiological plasticity mechanisms and led to further investigations of neuroplasticity in humans. In the neocortex, studies in animal models demonstrated a close association between motor learning and LTP-like plasticity (31–33). In humans, LTP-like plasticity was explored in the primary motor cortex (M1) concerning use-dependent plasticity (34–37), its involvement in motor learning (38), and its relevance for compensation of motor cortex dysfunctions after brain lesions (39). Post-transcriptional modifications of pe-existing protein account for LTP in the early phase, whereas alternations in the expression of genes and protein relate to LTP in the late phase (27). It has been shown that for studying the plasticity of the human brain, sensory inputs and non-invasive brain stimulation (NIBS) are able to alter respective cortical properties such as the strength of neural network connections, and movement representations (40, 41). Beyond its relevance to the learning formation of the healthy brain, cortical reorganization and adaptive plasticity apply to the field of neurorehabilitation (42–44).

## Multimodal therapies in rehabilitation

### Action observation treatment

Action observation and execution networks were found first in macaque monkeys. These networks are based on mirror neurons which are all-important to comprehending the actions of other individuals (45). The notion of mirror mechanisms displays that individuals observing an action could not only activate an identical or similar motor or motor-related cortical network but also automatically promote execution and motor skill acquisition in an observer (46, 47). Functional neuroimaging studies showed an observation-execution-dependent cortical network in human brains and revealed the overlapping of motor observation and motor execution in some brain regions. These networks incorporate M1, the primary somatosensory cortex, the ventral premotor cortex, several parietal areas, and the inferior frontal gyrus (48–52).

Respective observation-related network activation *via* observing a goal-directed movement of others promotes motor skill learning abilities and attainment of observers (53–55). Since long-term potentiation-like (LTP) plasticity is elevated by enhanced task-dependent motor cortex excitability (31, 56), the underlying mechanism of acquisition of a new motor skill *via* action observation might include LTP-like plasticity of these specific brain regions and network (57–60). Motor cortex activation by action observation might thus have the potential to develop into an effective rehabilitative strategy. In healthy humans, action observation enhances motor skill learning (46, 61–63), and action-related motor capacity with the untrained hand (64). AOT, in which action observation followed by execution of an identical task, has been used to alleviate motor function deficits in patients with neurological disorders (65). A typical rehabilitation session of AOT consists of an observation phase and an execution phase. A video clip of an actor and an actress performing object-directed daily action from different perspectives is presented on a computer screen. Specific action can be divided into three to four motor acts. Patients need to observe the motor act and execute the observed act afterwards (65, 66). In patients with acute ischemic stroke, AOT for 10 days facilitates relearning of upper extremity motor skills (67). For patients diagnosed with cerebral ischemic or hemorrhagic stroke in the subacute phase, AOT potentiated upper extremity motor function recovery, improved manual dexterity, and increased quality of life (68). AOT for 4 weeks improved upper extremity function and daily living performance in chronic stroke patients, and AOT of first-person perspective showed more beneficial effects in comparison with AOT of third-person perspective (69). AOT for 4 weeks has also been shown to promote gait ability in chronic stroke patients, and functional AOT was more effective than general AOT (70).

### Repetitive transcranial magnetic stimulation

TMS produces a time-varying magnetic field perpendicular to the stimulating coil, inducing electric currents in the cortical tissue beneath the scalp, and eliciting action potentials in targeted neuronal populations. As a neuromodulatory tool, repetitive TMS (rTMS) induces frequency-dependent after-effects. Low-frequency rTMS (LF-rTMS, ≤1 Hz) induces a prolonged decrease in cortical excitability, whereas high-frequency stimulation (HF-rTMS, ≥5 Hz) enhances cortical excitability (10, 71). Theta burst stimulation (TBS) is a subtype of rTMS, including intermittent (iTBS) and continuous (cTBS) stimulation that enhances and suppresses cortical excitability, respectively (72–74). HF-rTMS delivered to M1 concurrent with motor learning practice accelerated the rate of motor skill acquisition and improved motor performance in healthy individuals (75). It is assumed that the effect of this combined intervention is accomplished by the induction of LTP-like processes in the motor network, which promotes task-specific plasticity (75). In subacute hemorrhagic and ischemic stroke patients, delivery of HF-rTMS in the affected hemisphere facilitated motor function recovery of the paralytic hand (76). HF-rTMS over ipsilesional M1 promoted upper extremity motor recovery and daily living ability in acute stroke patients suffering from unilateral subcortical infarction in the middle cerebral artery (77). In subacute ischemic stroke patients, iTBS over the lesioned M1 prior to physiotherapy increased network connectivity between bilateral motor areas and M1, which is correlated with grip strength improvement (78). Resting-state interhemispheric motor network connectivity gradually decreases early after ischemic stroke and subsequently re-increases in the progress of motor function recovery (79). Application of iTBS facilitates reorganization of the motor network and induces neuronal plasticity, contributing to motor function recovery (78). It is proposed that HF-rTMS (76) and iTBS (78) over the ipsilesional M1 up-regulates the activity of the lesioned cortex. LF-rTMS applied over the unaffected motor cortex promoted motor function recovery and improved daily living ability in patients with cerebral infarction (80). LF-rTMS (80) and cTBS applied over the unaffected motor cortex down-regulates the excitability of the unaffected hemisphere and alleviates the interhemispheric inhibition imposed on the affected side. However, these approaches fail to induce beneficial effects in all stroke patients, and individuals respond differently to various stimulation parameters (81). Sankarasubramanian and co-workers demonstrated that upper limb reaching ability was facilitated by HF-rTMS over contralesional dorsal premotor cortex rather than standard stimulation approach (LF-rTMS over contralesional M1) in severely affected stroke patients (82). Therefore, classifying stroke patients into different subgroups (less affected vs. more affected) based on bimodal balance-recovery model is necessary for designing targeted and effective treatments.

### Transcranial electrical stimulation

Some studies showed that transcranial electrical stimulation (tES), including transcranial direct current (tDCS), transcranial random noise (tRNS), and transcranial alternating current (tACS) stimulation can increase the acquisition and retention of motor skills and improve motor functions in healthy humans, and rehabilitation (83, 84). These intervention tools elicit long-lasting augments or decrements of motor cortical excitability, and these effects are dependent on brain state and cognitive task performance before and/or during the intervention (85, 86).

tDCS modulates motor cortex excitability and/or activity *via* a weak electrical current (87), which de- or hyperpolarizes neuronal resting membrane potentials (86, 88). tDCS has a polarity-dependent influence on motor cortex excitability and/or activity. When the anode is positioned over M1, the amplitude of motor-evoked potentials (MEP) is increased (89, 90), whereas cathodal tDCS decreases MEPs with standard dosages (89, 91). Dependent on stimulation duration, tDCS can induce after-effects, which resemble LTP-like or LTD-like plasticity (85, 86, 92). In healthy humans, anodal tDCS over M1 during task execution improves motor learning (93–96). This effect is likely accomplished via modulation of LTP-like plasticity, and enhancement of functional connectivity of respective brain networks via anodal tDCS, resulting in motor performance improvement. Some studies reported that cathodal tDCS over M1 reduced motor performance speed (95, 97), but improved motor learning under specific conditions (98, 99). It is proposed that cathodal tDCS diminishes cortical excitability (“noise reduction”) via induction of LTD-like plasticity, thus focusing cortical activity on the neurons relevant to motor learning (93, 98, 99). In patient populations, this intervention has the potential to relieve maladaptive neuroplasticity and improve the neurophysiological state of the targeted brain regions as well as motor functions. The effects of tDCS on stroke patients were not consistently reported in different studies. Ojardias and co-authors reported that one session of anodal tDCS over ipsilesional M1 had a significant beneficial effect on gait endurance in chronic hemiplegic patients (100). In chronic ischemic and hemorrhagic stroke patients, two sessions of anodal tDCS applied over the lesioned M1 improved movement planning and preparation in a standing reaching task (101). Likewise, cathodal tDCS can also induce some positive effects in patients with stroke. Zimerman and co-works reported that cathodal tDCS applied to the non-lesioned M1 facilitated hand motor skill acquisition and retention in patients with subcortical ischemic stroke (102). Cathodal tDCS positioned over the unaffected motor cortex enhanced dual-task gait performance in chronic stroke patients (103). Seamon and co-works, however, indicated that neither anodal tDCS over the lesioned M1 nor cathodal tDCS over the non-lesioned M1 induced any significant effect on walking performance in chronic stroke patients (104). The variable effects of tDCS might be due to the inherent heterogeneity of the stroke patients, the variability of the stimulation parameters and the choice of motor paradigms (105). For stroke patients who benefit from tDCS, the interhemispheric balancing model has been proposed as the mechanism for motor function improvement. Anodal tDCS upregulates ipsilesional cortical excitability, improves network connectivity, and leads to alterations in interhemispheric balance (10). Cathodal tDCS over the contralesional M1 leads to downregulation of the contralesional cortical excitability and upregulation of the ipsilesional cortical excitability *via* reduced transcallosal inhibition (10, 106). Restoration of interhemispheric balance might be a relevant mechanism of tDCS-induced motor control improvement (107). As heterogeneity exists regarding the effect of tDCS on stroke patients, stratifying patients into different subgroups according to the etiology, the damage extent, and the phase of stroke is required to provide personalized therapeutic interventions.

tRNS is a relatively new neuromodulatory electrical stimulation method, which produces a white noise of a Gaussian or bell-shaped alternating current from 0.1 Hz to 640 Hz in a full-frequency spectrum or between 101 and 640 Hz in a high-frequency spectrum (108). Its random electrical oscillation spectrum in a full frequency spectrum or a high frequency spectrum applied to specific brain regions modulates neuronal membrane potentials, induces neuroplasticity, and results in an increase in motor cortex excitability (109–111). Proposed mechanisms of action are modulation of the neural signal-to-noise ratio *via* stochastic resonance (112, 113), and stimulation effects involve voltage-gated sodium channels (114–116). tRNS facilitates motor skill acquisition and consolidation in healthy humans (111, 117). Regarding the impact of tRNS in neurorehabilitation, Hayward and co-authors demonstrated that tRNS over ipsilesional M1 during reaching training improved clinical motor outcomes in chronic stroke patients suffering from severe arm dysfunction (118). tRNS combined with the Graded Arm Supplementary Program promoted upper extremity motor function recovery in ischemic stroke patients in the subacute phase (119). This implied that tRNS can boost functional adaptations of cortical tissue (118).

In tACS, another electrical non-invasive brain stimulation protocol, weak alternating sinusoidal currents over the cortical target region can entrain endogenous brain oscillations at some frequency brand (120). tACS enhanced either motor functions or cognitive functions via associated brain functions with stimulation frequencies matched to the natural dominant rhythm of the underlying brain area (121, 122). Antal et al. showed that tACS over M1 promoted motor learning in healthy humans (123). Beta-tACS over the lesioned M1 reduced the variance of sensorimotor beta-oscillations in stroke patients (124). With respect to motor rehabilitation, beta-tACS might be suitable for facilitating the specificity of brain self-regulation-based neurofeedback *via* interference with endogenous cortical rhythms and intrinsic brain oscillations in stroke patients (124).

In the human brain, regions are interconnected in complex functional networks, incorporating multiple anatomically remote but functionally interlinked areas (125–127). Some studies demonstrated that tES modulates brain activity and/or excitability in both local areas under the stimulation electrodes and remote interlinked brain regions (10, 128). Brain hubs have a critical impact on dynamic interactions between brain areas and integrate the information from different brain regions of the network (127, 129, 130). The effects of tES involving a node or hub of a specific cortical network can spread to functionally connected brain areas (128, 131, 132). Due to activity-dependent network models, tES-generated cortical activity and/or excitability alterations are furthermore sensitive to the specific state of brain networks, and dependent on the level of the ongoing activity of the stimulated cortical networks (128, 133). A wealth of studies has reported that tES can modulate behavior dependent on the neural activity level of brain networks involved in a task (98, 99, 134–137).

### Repetitive peripheral electrical and magnetic stimulation

Beyond non-invasive brain stimulation, peripheral stimulation techniques are also explored for their ability to improve neurorehabilitation. Non-invasive peripheral stimulation uses external devices to generate muscle contractions and sensory afferents that can be used in clinical settings to reduce pain and promote recovery of sensorimotor functions (138). Successful goal-directed movements necessary for interaction with the environment rely on the integration of sensory and motor information (139). Stroke is a common neurological disorder leading to compromised sensorimotor integration (140). Accurate sensorimotor integration of afferent and efferent signals in the cerebral cortex contributes to precise motor control and efficient action execution, and plays a critical role in motor learning. To target sensorimotor integration in stroke patients, either enhancement of afferent input to M1 by peripheral electrical stimulation (PES) or peripheral magnetic stimulation (PMS) to modulate motor output, or reduction of sensory input by temporary deafferentation, might be the potential therapeutic interventions (139).

PES activates not only superficial cutaneous receptors but also somatosensory nerve fibers (141, 142). PES over a muscle belly or a nerve at motor threshold intensity induces muscle contractions by depolarization of motor axons and facilitates motor unit recruitment (143). Modulation of afferent input by PES at motor threshold induces neuroplastic alternations and organizational changes in the sensorimotor cortex, and increases cortical excitability that produces adaptations in central motor pathways (144–147). PES over a nerve at sensory threshold intensity enhances somatosensory input, improves corticomotor excitability (148), facilitates connectivity in sensorimotor regions (149), and induces reorganization of cortical maps (150). Some studies reported that PES improves motor learning (151), motor memory consolidation (152), and inter-limb transfer of motor skills (149) in healthy individuals. In stroke patients, PES at motor threshold increased wrist range of motion and hand muscle strength, improved muscle tone and muscle electrical activity, enhanced functional performance of the upper extremity, and promoted daily living capacity (153, 154). In patients with subacute and chronic stroke, PES at motor threshold decreased muscle spasticity, increased muscle strength, facilitated gait performance, and promoted motor function recovery of the lower extremity (155, 156). One session of PES at sensory threshold reduced muscle spasticity, enhanced muscle strength and proprioception, and improved balance and gait ability in chronic stroke patients (157–159).

In comparison to PES, PMS is deemed to stimulate deeper tissue regions and induce strong muscle contractions for neuromuscular stimulation, with less pain, and fewer side effects with respect to stimulation of the spinal root, muscle belly, or nerve (160, 161). PMS increases peripheral venous blood flow (162), induces muscle contractions with minimal cutaneous sensations (138), and reduces spasticity and muscle hyperreflexia (163). PMS effects depend on the induction of the activity of proprioceptive afferents to the central nervous system, which results in modulation of the excitability of specific spinal circuits and the motor cortex (142, 164–166). PMS improved motor functions in healthy humans (167). In stroke patients, PMS can also induce some beneficial effects. It is reported that in patients with severe upper extremity paresis during the early acute and subacute phase of stroke, PMS prior to standard care promoted upper limb functions, improved daily living abilities, and accelerated the progress rate of motor function recovery (168, 169). In chronic stroke patients with ankle impairment, PMS improved ankle joint mobility and muscle strength, increased M1 transsynaptic excitability in the contralesional hemisphere, and decreased short-interval intracortical inhibition in both hemispheres (170, 171). It is hypothesized that proprioceptive afferents generated by PMS reduce GABAergic inhibition, and the induction of brain plasticity in the sensorimotor cortex may contribute to the increase of muscle strength (171). Furthermore, a single session of PMS significantly reduced spasticity along with decreased event-related desynchronization of mu rhythm in the contralesional hemisphere in subacute or chronic stroke patients (172). It is proposed that the reduction of spasticity might be related to cortical activity alternations in the contralesional hemisphere (172).

### Combined intervention therapies in neurorehabilitation

Action observation treatment, transcranial electrical or magnetic stimulation, and peripheral electrical or magnetic stimulation are important components for the development of new treatment methods in the field of neurorehabilitation.

The combined intervention of NIBS and action observation can modulate neuroplasticity and motor functions in both healthy and stroke patients. Our previous studies showed that tRNS over M1 paired with mirror-matching action observation enhances observation-dependent motor cortex excitability, and then this effect promotes execution-dependent motor cortex excitability (137). Some studies reported that action observation improves connectivity between the ventral premotor cortex and M1, and movement execution promotes connections either between the dorsal premotor cortex and M1 or the supplementary motor region and M1 (55, 173). tRNS and motor observation might have synergistic effects in improving cortical excitability *via* premotor mirror neurons to directly and/or indirectly activate M1 neurons. Vice versa, 20 Hz tACS with target electrode over the left M1 and return electrode over the contralateral supraorbital region during movement observation inhibits motor cortex excitability and subsequently inhibits action execution-dependent cortical excitability (174). As a neurophysiological biomarker of functional reorganization, suppression of beta power oscillations is associated with motor learning and consolidation (175). These findings indicated that action observation combined with TES resulted in changes of task-dependent motor cortex activity, which could be advantageous to prevent pathological alterations in stroke sickness (65). In stroke patients with ideomotor apraxia, AOT combined with LF-TMS over the intact hemisphere increased motor cortex excitability and facilitated the recovery of hand motor function (176). LF-TMS over contralesional M1 during observation of complex hand movements improved distal upper extremity functions in the subacute phase following stroke (177). Action observation coupled with PES induced a long-lasting increase in primary motor cortex excitability (178) and improved spontaneous movement tempo (179) in healthy persons. It is proposed that PES paired with action observation might be a promising treatment technique in neurorehabilitation. PES is thought to provide movement-related afferent stimulation to consolidate the kinematic information learned from action observation and lead to neuroplastic adaptations (179).

Some studies explored the effects of transcranial magnetic or electrical stimulation combined with peripheral electrical or magnetic stimulation techniques in neurorehabilitation. In healthy individuals, the effects of combined brain and peripheral stimulation were inconsistently reported. Anodal tDCS (1 mA) alone for 5 min transiently increased cortical excitability, whereas anodal tDCS paired with PES prolonged the facilitating effect for up to 60 min (180). Likewise, cathodal tDCS (1 mA) alone for 5 min decreased the cortical excitability immediately after the stimulation, and the changes were prolonged for up to 60 min when combined with PES (180). The proposed mechanism is that anodal tDCS paired with PES induces LTP-like plasticity and cathodal tDCS combined with PES evoked LTD-like plasticity (180). Schabrun and co-authors, however, failed to find any summative effects after concurrent application of 1 mA tDCS and peripheral nerve electrical stimulation for 20 min, which might be explained by the homeostatic plasticity mechanism (181). In another study, 2 mA anodal tDCS significantly increased MEP amplitude, whereas tDCS combined with PES did not induce any changes in MEP amplitude, indicating a suppression effect following combined stimulation (182). In patients within the first few days following a stroke, anodal tDCS over the ipsilesional M1 coupled with PES of the paretic hand for 5 consecutive days promoted hand motor function recovery (183). In chronic stroke patients, tDCS over the ipsilesional M1 combined with PES prior to motor training potentiated the beneficial effects of motor learning beyond levels reached with tDCS or PES alone (184). This might be that tDCS paired with PES produces additive effects on motor functions through different pathways where anodal tDCS depolarizes neuronal membrane potential and modulates Glutamate as well as GABA concentrations (86, 185), whereas PES modulates GABAergic interneurons activity (184, 186). In contrast, Menezes and co-authors reported that one session of combined stimulation (PES of the paretic arm and tDCS over the ipsilesional M1) prior to motor training did not facilitate training effects on range of motion, gasp and pinch strength in chronic stroke patients with moderate to severe upper extremity motor deficits (187). As discrepancy exists, more studies are needed to optimize the simulation parameters to induce the beneficial effects of this combined intervention. Paired associative stimulation (PAS) modulates motor cortex excitability based on associative LTP/LTD mechanism governed by Hebbian principles (188–190). When PES was applied 10 ms prior to TMS, motor cortex excitability was increased (facilitatory PAS), whereas motor cortex excitability was inhibited when PES is delivered 25 ms preceding TMS (inhibitory PAS) (190). Facilitatory PAS enhanced motor learning in healthy humans (191). It is suggested that PAS induces LTP-like plasticity, and triggers alterations in synaptogenesis and structure connectivity, leading to the facilitation of motor learning (191). Furthermore, facilitatory PAS can promote motor functions in stroke patients *via* the upregulation of motor cortex excitability in the ipsilesional hemisphere (192). Other forms of associative stimulation, though with limited investigations, showed some promise in treating neurological diseases. Kumru et al. reported that repetitive TMS at 0.1 Hz combined with rPMS at 10 Hz increased motor cortex excitability and reduced intracortical inhibition that might be mediated by GABA-ergic inhibition, but repetitive TMS at 0.1 Hz or rPMS at 10 Hz, respectively, did not improve motor cortex excitability (193).

**Figure 1:**
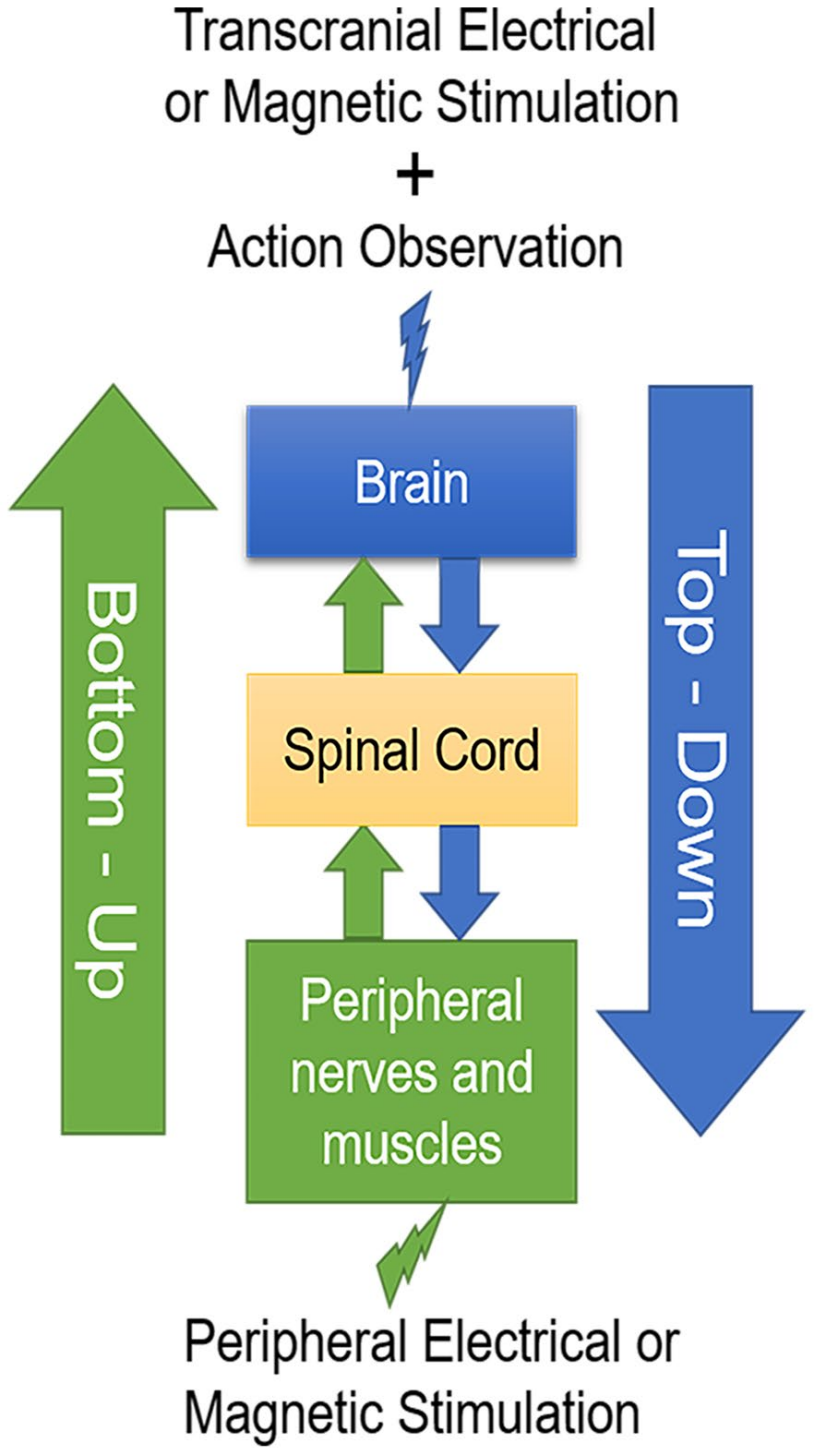
A schematic drawing of the two-stage stimulation model. Transcranial electrical or magnetic stimulation during action observation in a top-down manner combined with peripheral electrical or magnetic stimulation in a bottom-up manner develop into valuable physical therapy strategies in neurorehabilitation after stroke.

Both central and peripheral stimulation protocols modulate cortical activity in a state-dependent manner (194–197). The cortical activity in action observation and execution network can be modulated by AOT and synchronously central and peripheral stimulation techniques. Combined top-down with bottom-up stimulation approaches could synergistically modulate cortical activity, spinal networks as well as motor unit recruitment in muscle, reduce spasticity and muscle hyperreflexia, and develop into physical therapy strategies in neurorehabilitation of stroke patients (Figure 1).

Both top-down and bottom-up stimulation techniques have shown some promise in promoting stroke recovery. However, as studies vary in the extent of the structural reserve, the simulation parameters, the phase of stroke, the duration of follow-up, and the outcome measurements, the therapeutic efficacy of different simulation techniques are inconsistently reported. The existing evidence is insufficient to make clinical recommendations in different phases post-stroke, and the way to appropriately apply these techniques in the clinical setting remains to be clarified. Top-down and bottom-up stimulation combined with AOT may have synergistic effects to reach a clinically meaningful level in stroke patients, which need to be investigated in well-designed randomized controlled trial studies with prolonged follow-up.

There are a few limitations that should be mentioned in this perspective. First, we did not differentiate the results following the time windows post-stroke. The neuromodulating effect of variable techniques may change in different stages of stroke. In addition, we did not discuss other neurorehabilitation approaches such as mirror therapy, motor imagery and constraint-induced movement therapy. Last, we did not include other new forms of neuromodulation techniques for instance vagal nerve stimulation and extremely low-frequency magnetic fields (11).

## Conclusion and future perspectives

Functional recovery after a stroke depends on the extent of structural reserve of the lesioned hemisphere. The interhemispheric competition model dominates in stroke patients with high structural reserve, whereas the vicariation model dominates in those with little structural reserve. In line with this bimodal balance-recovery model, future studies should explore the effects of (1) anodal tDCS, beta tACS, high-frequency tRNS, HF-TMS, or iTBS over the contralesional hemisphere combined with PES or PMS of the paretic limbs during motor observation followed by motor execution of an identical task on subsequent motor execution-dependent motor cortex excitability in the stroke patients of the severe lesioned hemisphere; (2) anodal tDCS, beta tACS, HF-tRNS or HF-TMS over the lesioned hemisphere and cathodal tDCS, LF-TMS, or cTBS over the non-lesioned hemisphere combined with PES or PMS of the paretic limbs during motor observation followed by motor execution of an identical task on subsequent motor execution-dependent motor cortex excitability in stroke patients with high structural reserve of the lesioned hemisphere. Further research also considers its feasibility for recovery of motor functions in upper and lower limbs in stroke patients. The combination of these techniques followed by motor execution may have a synergic effect to optimize neuroplastic changes and improve motor recovery. The task-dependent neuronal network might be efficiently connected when participants observed the correspondingly complex movement under the combination stimulation techniques, which then promoted task-dependent network activity during performance of the identical task. The proposed paradigms are an innovative approach and could be an adjunctive therapy to potentiate the effect of conventional rehabilitation treatment, especially for those patients with severe motor deficit. Future studies are required to improve the efficacy of the respective interventions, and to validate these results in larger multicenter clinical trials.

## Author contributions

FQ, MN, LW, and DW contributed to the conception and design. FQ, LW, and DW drafted the paper. MN and XR revised it critically for important intellectual content. All authors have read and agreed to the published version of the manuscript.

## Funding

This research was funded by the Special Fund for Fundamental Research Funds of the Central Universities (Sports Rehabilitation Science Laboratory), the Fundamental Research Funds for the Central Universities (2022QN001), and the Research Foundation for Advanced Talents of Beijing Sport University.

## Conflict of interest

MN is member of the scientific advisory board of Neuroelectrics.

The remaining authors declare that the research was conducted in the absence of any commercial or financial relationships that could be construed as a potential conflict of interest.

## Publisher’s note

All claims expressed in this article are solely those of the authors and do not necessarily represent those of their affiliated organizations, or those of the publisher, the editors and the reviewers. Any product that may be evaluated in this article, or claim that may be made by its manufacturer, is not guaranteed or endorsed by the publisher.
